# The Potential MicroRNA Diagnostic Biomarkers in Oral Squamous Cell Carcinoma of the Tongue

**DOI:** 10.3390/cimb46070402

**Published:** 2024-07-01

**Authors:** Young-Nam Park, Jae-Ki Ryu, Yeongdon Ju

**Affiliations:** 1 Department of Dental Hygiene, Gimcheon University, Gimcheon 39528, Republic of Korea; ivy9797@empas.com; 2 Department of Biomedical Laboratory Science, Gimcheon University, Gimcheon 39528, Republic of Korea; rs0429@daum.net

**Keywords:** oral squamous cell carcinoma, microRNA, diagnosis, biomarker

## Abstract

Oral squamous cell carcinoma (OSCC) of the tongue is a common type of head and neck malignancy with a poor prognosis, underscoring the urgency for early detection. MicroRNAs (miRNAs) have remarkable stability and are easily measurable. Thus, miRNAs may be a promising biomarker candidate among biomarkers in cancer diagnosis. Biomarkers have the potential to facilitate personalized medicine approaches by guiding treatment decisions and optimizing therapy regimens for individual patients. Utilizing data from The Cancer Genome Atlas, we identified 13 differentially expressed upregulated miRNAs in OSCC of the tongue. Differentially expressed miRNAs were analyzed by enrichment analysis to reveal underlying biological processes, pathways, or functions. Furthermore, we identified miRNAs associated with the progression of OSCC of the tongue, utilizing receiver operating characteristic analysis to evaluate their potential as diagnostic biomarkers. A total of 13 upregulated miRNAs were identified as differentially expressed in OSCC of the tongue. Five of these miRNAs had high diagnostic power. In particular, miR-196b has the potential to serve as one of the most effective diagnostic biomarkers. Then, functional enrichment analysis for the target gene of miR-196b was performed, and a protein–protein interaction network was constructed. This study assessed an effective approach for identifying miRNAs as early diagnostic markers for OSCC of the tongue.

## 1. Introduction

Oral squamous cell carcinoma (OSCC) of the tongue is the most common cancer in the squamous cells of the tongue and is marked by an insidious nature [1]. It represents a significant portion of head and neck squamous cell carcinoma (HNSCC) cases and accounts for approximately 31.9% of oral cavity cancers [2,3]. The main risk factors associated with the development of tongue cancer are tobacco smoking and excessive alcohol consumption. The early symptoms of OSCC are foreign body sensation or swallowing pain [4]. The treatment of tongue cancer is contingent upon the staging and anatomical site of the tumor, typically necessitating an approach encompassing surgery, radiation therapy, and chemotherapy. Persistent investigation into early detection modalities and precision therapies is imperative for enhancing the therapeutic outcomes for individuals afflicted with tongue cancer. OSCC is one of the most diagnosed malignancies and a prominent contributor to mortality attributed to head and neck cancers [5]. OSCC has a poor prognosis because of the lack of a strong barrier for preventing tumor propagation [6]. Anticipating the prognosis of individuals diagnosed with OSCC holds importance in devising treatment strategies. In the early stage of OSCC, which is marked by a favorable prognosis, cancer-related mortality affects approximately 19% of patients [7]. The early detection of cancer correlates with elevated survival rates and diminished healthcare expenditures among patients, owing to decreased dependence on aggressive therapeutic modalities [8]. Early detection and treatment can greatly improve the prognosis for patients with OSCC [9].

MicroRNAs (miRNAs) are short noncoding RNAs that play crucial roles in the regulation of gene expression and comprise approximately 22 nucleotides [10,11]. miRNAs operate by binding to messenger RNA molecules, thereby either inhibiting translation initiation or degrading mRNA [12]. miRNAs exhibit differential expression, being either upregulated or downregulated and are associated with the status and progression of tumors [13]. Several miRNAs have enhanced potential as biomarkers for diagnosing or monitoring specific cancers because they demonstrate cancer-specific expression patterns [14]. miRNA plays a crucial role in understanding the biological processes of tumors and developing treatment strategies.

The Cancer Genome Atlas (TCGA) resource serves as a platform for the diagnosis, prognosis, and immunotherapy of cancer, including the exploration of potential miRNA-based biomarkers [15,16,17]. Graphical representations such as heatmaps and volcano plots facilitate the visualization of miRNA expression patterns and distinguish differential expression profiles. The objective of our study was to identify certain miRNAs essential for diagnosing OSCC by employing a methodology based on TCGA data. This approach, which includes diagnostic tools, discriminates between malignant and adjacent nontumorous tissues with remarkable sensitivity and specificity. Among the myriad candidates scrutinized, five miRNAs emerged as promising contenders for OSCC diagnosis, emphasizing their potential as biomarkers to augment the clinical management of tongue cancer, particularly in the early stages. Our investigation prioritized the exploration of biomarkers aimed at discerning OSCC. Utilizing bioinformatics, we performed a comprehensive analysis of OSCC transcriptome data, aiming to elucidate the molecular mechanisms underlying OSCC development and to identify specific molecules critical for its progression.

## 2. Materials and Methods

### 2.1. Acquisition of TCGA Data

The miRNA expression profiles of OSCCs were downloaded from the TCGA data portal (http://firebrowse.org/, accessed on 3 May 2024), comprising 128 tumor samples and 13 adjacent nontumorous tissue samples. OSCC expression profiles were extracted from the TCGA HNSCC dataset, with the primary tumor site filtered to include only those of the tongue within the HNSCC dataset. Each sample comprised 1046 miRNA expression values obtained through the Illumina HiSeq platform. The miRNA expression profiles were generated using miRNA sequencing data, which represented a record of the reads per million miRNAs that were mapped. Clinical information regarding OSCC was sourced from the TCGA data portal. Table 1 presents comprehensive characteristics of the study, including genders, ages, pathologic stages, and tumor-node-metastasis classifications.

### 2.2. Selection of Candidate Diagnostic miRNAs

Identifying miRNAs as candidate biomarkers provides an important approach for advancing cancer diagnosis. Due to their stability and specificity, miRNAs are excellent diagnostic tools. Their utilization can lead to the development of accurate and sensitive diagnostic assays. By identifying cancer-specific miRNAs, researchers can improve early detection and treatment strategies for individual cancer patients. Heatmap analysis and volcano plots serve as valuable tools in elucidating discernible patterns of miRNA expression [18,19]. The receiver operating characteristic (ROC) curves can provide evaluations of the accuracy of the predictive model selection of biomarkers [20]. The area under the curve (AUC) is a fundamental metric obtained from the ROC curve, offering a holistic evaluation of a diagnostic test’s performance. It quantifies the ability of a diagnostic test to discriminate between disease cases and non-cases [21]. A higher AUC indicates better diagnostic performance [22]. In studies evaluating diagnostic value, an AUC exceeding 0.90 indicates excellent diagnostic performance of the test [23]. We selected differentially expressed miRNAs, which were analyzed using a heatmap and a volcano plot. Subsequently, ROC curves were generated, and the AUC was calculated with a 95% confidence interval (95% CI). ROC analysis identifies the optimal cutoff point, where sensitivity and specificity are maximized, for diagnostic biomarker values [24]. The point on the curve that maximizes the Youden Index is selected as the optimal threshold [24]. Diagnostic sensitivity and specificity were computed using GraphPad Prism software 6.0. miRNAs exhibiting an AUC > 0.9 were identified as potential diagnostic biomarkers.

### 2.3. Prediction of microRNA Targets

Predicting the target genes of miRNAs is crucial for regulating gene expression within cells and comprehending their involvement in the progress of cancers [25]. Studying miRNA–target interactions could reveal the biological function of certain miRNAs. We identified potential target mRNAs through the online miRNA prediction database miRDB (https://mirdb.org/, accessed on 8 May 2024). miRDB is a database for miRNA target prediction and functional annotation [26].

### 2.4. Functional Enrichment Analysis

Functional enrichment analysis is a bioinformatics computational method utilized for the interpretation of large-scale omics data, such as gene expression profiles or genomic datasets. Functional enrichment analysis assists researchers with uncovering the underlying biological mechanisms associated with observed experimental results. Gene ontology (GO)-based approaches utilize functional annotations to predict cancer driver genes [27]. The GO provides a hierarchical framework of terms organized into three main categories, including biological process, molecular function, and cellular component. Each term within GO represents a specific biological activity, contributing to a comprehensive understanding of gene functions and cellular organization. GO analysis was conducted using the DAVID online tool (https://david.ncifcrf.gov/, accessed on 10 May 2024). DAVID is a widely used bioinformatics resource system, comprising a web service for functional enrichment analysis and annotation [28]. The Kyoto Encyclopedia of Genes and Genomes (KEGG) is a comprehensive database and resource that links genomic information to higher-order functional information [29]. A *p* < 0.05 was considered to indicate statistical significance. KEGG provides a suite of tools dedicated to pathway enrichment analysis, facilitating the identify of biological pathways related to a set of genes or proteins.

### 2.5. Protein–Protein Interaction Network

Protein–protein interaction (PPI) networks facilitate the identification of diagnostic and prognostic biomarkers in cancer research by elucidating altered protein interactions within cancer cells [30]. PPI network exploration utilized the Search Tool for Retrieval of Interacting Genes/Proteins (STRING) database, a widely acknowledged resource for protein interaction data analysis (https://string-db.org/, accessed on 27 May 2024) [31]. Visualization of the network analysis was accomplished using Cytoscape software 3.9.1. CytoHubba was used to score node genes using the maximum clique centrality (MCC) algorithm [32].

### 2.6. Statistical Analyses

Data analysis and visualization were conducted using GraphPad Prism software 6.0 (GraphPad Software, San Diego, CA, USA). The independent samples t-test was used to determine statistical differences between groups. ROC curve analysis was employed, and the AUC was calculated to assess the diagnostic significance. A significance threshold of *p* < 0.05 was applied for statistical interpretation.

## 3. Results

### 3.1. Identification of Differentially Expressed miRNAs in OSCC Patients from TCGA Database

**Figure 1:**
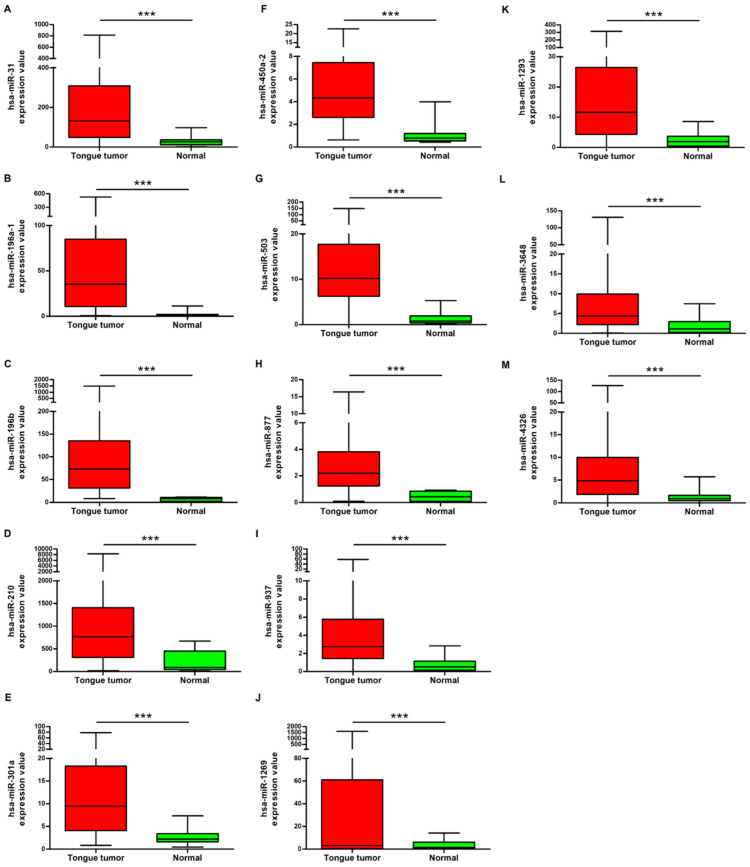
Relative expression levels of 13 differentially expressed miRNAs in oral squamous cell carcinoma of the tongue tissue and non-tumor tissue. (**A**) miR-31; (**B**) miR-196a-1; (**C**) miR-196b; (**D**) miR-210; (**E**) miR-301a; (**F**) miR-450a-2; (**G**) miR-503; (**H**) miR-877; (**I**) miR-937; (**J**) miR-1269; (**K**) miR-1293; (**L**) miR-3648; (**M**) miR-4326. *** *p* < 0.001.

**Figure 2:**
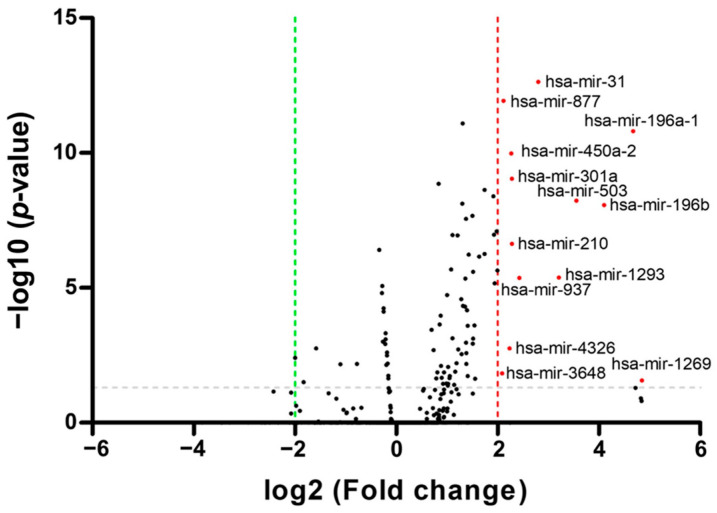
Volcano plot for the identification of differentially expressed miRNAs. A volcano plot was used to indicate the log2 fold change in 13 differentially expressed miRNAs. Differentially expressed miRNAs are ranked based on fold change and *p*-value. Adjustment for multiple testing was performed using Bonferroni correction, with a significance threshold set at *p* < 0.01.

A differential expression analysis was performed to identify miRNA expression levels that exhibited significant differences between OSCC tissues and adjacent nontumorous tissues (Figure 1). TCGA data were utilized for this analysis, which contrasted miRNA expression patterns in OSCC patients with those in HNSCC patients. A total of 13 upregulated miRNAs were identified as differentially expressed through heatmap analysis, with visualization facilitated by MultiExperiment Viewer 4.9.0 (Figure S1). Differentially expressed miRNAs with a *p*-value less than 0.05 and significant log2 fold changes were identified and visualized on the volcano plot [33]. The volcano plot was created using GraphPad Prism 6.0. (Figure 2).

### 3.2. Evaluation of the Diagnostic Values of the Five Potential miRNAs

Among the thirteen upregulated miRNAs, the AUC values (with a 95% CI) of hsa-miR-196a-1, hsa-miR-196b, hsa-miR-450a-2, hsa-miR-503, and hsa-miR-877 were 0.9447 (0.8991–0.9903), 0.9838 (0.9661–1.001), 0.9303 (0.8588–1.002), 0.9549 (0.9153–0.9946), and 0.9285 (0.8791–0.9779), respectively (Figure S2). These AUC values indicate the discriminative ability of each miRNA in distinguishing between OSCC tissues and adjacent nontumorous tissues, with higher values indicating better diagnostic efficacy. Notably, miR-196b exhibited the highest diagnostic value among the miRNAs evaluated. Detailed diagnostic values for five differentially expressed miRNAs are presented in Table 2.

### 3.3. Gene Ontology and KEGG Pathway Enrichment Analysis

We conducted a custom prediction search for the 369 target genes of miR-196b-5p and the 48 target genes of miR-196b-3p using miRDB. The results of the target genes for miR-196b-5p and miR-196b-3p are presented in Table S1. Furthermore, we utilized the DAVID database to identify potential biological functions of the target genes of miR-196b-5p and miR-196b-3p through GO enrichment analyses. The biological process group of the target genes of miR-196b-5p consisted of positive regulation of transcription from RNA polymerase II (GO:0045944), embryonic skeletal system morphogenesis (GO:0048704), and anterior/posterior pattern specification (GO:0009952). The biological process group of the target genes of miR-196b-3p included RNA 3′ uridylation (GO:0071076), positive regulation of 3′-UTR-mediated mRNA stabilization (GO:1905870), and a defense response to Gram-negative bacteria (GO:0050829). The cellular component group of the target genes of miR-196b-5p comprised nucleoplasm (GO:0005654), nucleus (GO:0005634), and cytoplasmic stress granule (GO:0010494). The cellular component group of the target genes of miR-196b-3p included cytoplasm (GO:0005737), cilium (GO:0005929), and cytosol (GO:0005829). The molecular function group of the target genes of miR-196b-5p included transcriptional activator activity, RNA polymerase II transcription (GO:0001228), DNA binding (GO:0003677), and chromatin binding (GO:0003682). The molecular function group of the target genes of miR-196b-5p consisted of calcium ion binding (GO:0005509) and lipopolysaccharide binding (GO:0001530). GO analysis of target genes is shown in Table S2. The KEGG pathway was mainly enriched in axon guidance (hsa04360), spinocerebellar ataxia (hsa05017), the MAPK signaling pathway (hsa04010), the Ras signaling pathway (hsa04014), the mRNA surveillance pathway (hsa03015), mitophagy (hsa04137), and protein digestion and absorption (hsa04974). The results of KEGG enrichment analysis are shown in Table S3.

### 3.4. Identification of the Hub Genes

**Figure 3:**
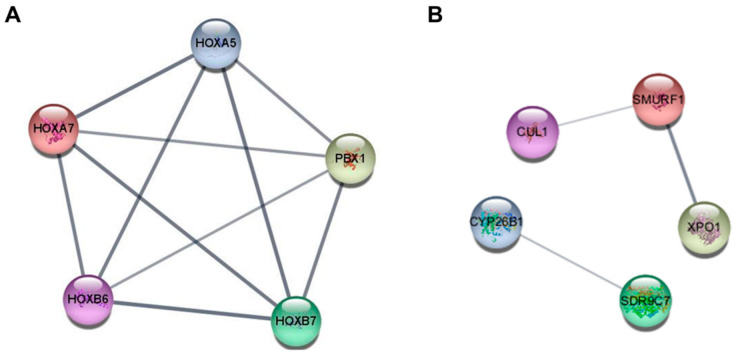
A protein–protein interaction network of the top five hub genes. (**A**) The top five hub genes of miR-196b-5p. (**B**) The top five hub genes of miR-196b-3p.

The identification of hub genes in cancer is essential for elucidating the intricate molecular mechanisms that drive cancer development, progression, and responses to treatment. Hub genes serve as nodes within gene regulatory networks and signaling pathways implicated in cancer pathogenesis. The identification of hub genes plays a critical role in advancing our understanding of cancer biology. The STRING database was utilized to construct and analyze a PPI network, with the minimum correlation coefficient threshold set at 0.400. The top five hub genes of miR-196-5p in the PPI network were identified using the MCC algorithm implemented in CytoHubba. The identified hub genes include Homeobox A5 (HOXA5), Homeobox A7 (HOXA7), Homeobox B6 (HOXB6), Homeobox B7 (HOXB7), and PBX Homeobox 1 (PBX1) (Figure 3A). The top five hub genes of miR-196-3p were SMAD specific E3 ubiquitin protein ligase 1 (SMURF1), cullin 1 (CUL1), exportin 1 (XPO1), cytochrome P450 family 26 subfamily B member 1 (CYP26B1), and short-chain dehydrogenase/reductase family 9C member 7 (SDR9C7) (Figure 3B).

## 4. Discussion

Biomarkers represent a promising avenue for advancing personalized medicine and optimizing therapy tailored to individual patients, while concurrently facilitating the exploration of novel drug targets and the development of novel treatment strategies [34]. Diagnostic biomarkers play a crucial role in either detecting or confirming the presence of specific diseases or conditions [35]. In clinical applications, the effective utilization of miRNAs as diagnostic biomarkers necessitates adherence to stringent criteria, notably high sensitivity and specificity. Additionally, an ideal biomarker for a specific cancer type should exhibit significant differential expression [36]. Ultimately, biomarkers hold the potential to elevate standards of patient care and drive precision medicine initiatives forward [37]. miRNA profiling has garnered considerable attention as a valuable tool for tumor classification, early detection, disease prognosis, and therapeutic decision making. However, despite notable advancements, the field of miRNA research encounters persistent technical hurdles. The costs associated with miRNA profiling are still high and serve as a barrier to adoption. However, the utilization of miRNAs as biomarkers offers several advantages, including their manageable dimensionality, ease of testing, and clinical significance in disease diagnosis [38]. Detection of a select few miRNAs provides deeper insights into tumor developmental lineage and differentiation compared to profiling numerous mRNAs [39].

Diagnostic biomarkers for tongue cancer include various molecular entities such as long intergenic non-coding RNA, miRNAs, and metabolites. These biomarkers show promise for the early detection and monitoring of oral cancer. In previous research, the miRNA expression profiles of oral squamous cell carcinoma (OSCC) were investigated [40,41]. Salivary LINC00657 and miRNA-106a have been identified as potential diagnostic markers for OSCC [42]. Additionally, N-acetyl-D-glucosamine, L-pipecolic acid, and L-carnitine have been investigated as the signature diagnostic biomarkers for OSCC [43]. In this study, we identified 13 miRNAs that were differentially expressed in OSCC patients. The findings from this research demonstrate a notable upregulation in the expression of hsa-miR-31, hsa-miR-196a-1, hsa-miR-196b, hsa-miR-210, hsa-miR-301a, hsa-miR-450a-2, hsa-miR-503, hsa-miR-877, hsa-miR-937, hsa-miR-1293, hsa-miR-3648, and hsa-miR-4326 within tissue samples obtained from patients diagnosed with OSCC in comparison to control samples. Subsequently, through ROC analysis, we identified these selected miRNAs as diagnostic biomarkers of OSCC. Among these, five miRNAs demonstrated an AUC value of 0.9 or higher, with miR-196b emerging as particularly noteworthy in terms of diagnostic significance. The expression levels of miR-196a-1 are known to be markedly upregulated in tissue and plasma samples derived from individuals diagnosed with colorectal cancer compared with controls [44]. miR-450a-2 exhibited downregulation in both gastric cancer cells and tissue [45]. The downregulation of miR-503 leads to an inhibition in the progression in OSCC [46]. miR-877-3p targets vascular endothelial growth factor A (VEGFA), and the positive expression of VEGFA has been associated with a significantly poor prognosis In cases of OSCC [47]. miR-196b has been proposed as a potential biomarker for the management of oral cancer, and its overexpression has been associated with enhanced oral cancer cell migration, invasion, and lymph node metastasis [48]. Elevated expression levels of miR-196a and miR-196b have been observed in saliva samples from patients diagnosed with HNSCC. These findings highlight the potential of these miRNAs as diagnostic biomarkers for detecting HNSCC at an early stage [49]. Additionally, the combined determination of plasma miR-196a and miR-196b may be used as a diagnostic biomarker for the early detection of oral cancer [50].

Identifying target genes can aid in the development of personalized therapies based on genetic profiles. Predicted target genes of miR-196b were categorized based on their biological processes, cellular components, and molecular functions using GO analysis. Furthermore, analysis of enriched KEGG pathways has been conducted to elucidate the involvement of target genes of miR-196b in oral cancer. The expression of axon guidance genes is related to clinical features like pain and nodal status in oral cancer [51]. Clinical studies have reported dysphagia in patients with spinocerebellar ataxia type 2, type 3, type 6, and type 7 [52]. Dysphagia in oral cancer is commonly attributed to extensive tissue destruction, limited excursion of the remaining tissue, and sensory paralysis of the tongue [53]. The MAPK signaling pathway plays a crucial role in OSCC, where it interacts extensively with miRNAs to regulate cellular processes involved in OSCC development and progression [54]. The Ras signaling pathway is intricately linked to the pathogenesis of OSCC and contributes to key oncogenic processes such as cell proliferation, invasion, and metastasis [55]. The role of mRNA surveillance pathways, notably the nonsense-mediated mRNA decay (NMD) pathway, have been implicated in the pathogenesis of cancer. Additionally, the NMD pathway functions as a post-transcriptional regulator [55]. Mitophagy is a cellular process that selectively degrades dysfunctional mitochondria through autophagy [56]. Targeting mitochondria may be a promising way to treat OSCC [57]. Protein digestion and absorption are associated with oral cancer due to their role in nutrient uptake and cellular metabolism [58]. The top five hub genes of miR-196b-5p were screened using the MCC algorithm. These genes include HOXA5, HOXA7, HOXB6, HOXB7, and PBX1. Altered expression patterns of homeobox-containing HOX genes have been implicated in oral cancer [59]. The expression of HOXA5 was upregulated in OSCC samples compared to non-tumor tissue and was associated with survival rates [60]. Expression of HOXA7 in OSCC exhibited a substantial increase at both the mRNA and protein levels [61]. H3OXB6 was hypermethylated in OSCC cell lines (SCC4 and SCC9) derived from a human HNSCC [62]. HOXB7 is implicated in abnormal proliferation in oral carcinogenesis [63]. Expression analysis demonstrated the expression of PBX1 mRNA and protein within OSCC cells [64]. The top five hub genes of miR-196b-3p were SMURF1, CUL1, XPO1, CYP26B1, and SDR9C7. While this study presents promising findings, it is important to acknowledge several limitations. Conducting a larger cohort study is essential to further validate these findings. Additionally, investigating the functional roles of miRNAs through in vitro and in vivo studies in future research would significantly deepen our understanding of their biological mechanisms and signaling pathways. Nevertheless, our findings reveal a significant upregulation of miR-196b in tissues from oral cancer patients, suggesting its potential utility as a diagnostic biomarker.

## 5. Conclusions

A current method of diagnosing and screening OSCC of the tongue is the scalpel biopsy, which is time consuming and requires considerable expertise. While advanced imaging modalities such as computed tomography (CT) and magnetic resonance imaging (MRI) technologies have advanced significantly in recent decades, CT scans can only detect the presence of masses [65], thus underscoring the need for supplementary diagnostic tools. The differential expression patterns of miRNAs in cancerous tissue samples and adjacent nontumorous tissue samples serve as valuable biomarkers for diagnostic purposes [66]. In conclusion, this study elucidated a five-miRNA diagnostic model associated with patients diagnosed with OSCC of the tongue. Our findings highlighted the remarkable diagnostic potential of miR-196b. Additionally, five hub genes were selected among the target genes of miR-196b. Ensuring the stability and reproducibility of biomarkers is crucial for augmenting their clinical applicability. Further research will prioritize the standardization of biomarker measurement methodologies and validation of biomarker performance.

## Figures and Tables

**Table 1:** Clinical data of patients with oral squamous cell carcinoma of the tongue from The Cancer Genome Atlas.

Characteristic	Overall
Gender, *n* (%)	
Male	82 (64.1)
Female	46 (35.9)
Age (years)	
Mean ± SD	58.17 ± 13.27
NA	1
Pathologic stage, *n* (%)	
Stage I	15 (11.72)
Stage II	22 (17.19)
Stage III	30 (23.44)
Stage IV	51 (39.84)
Not Available	10 (7.81)
Pathological, T, *n* (%)	
T1	22 (17.19)
T2	45 (35.16)
T3	34 (26.56)
T4	19 (14.84)
TX	6 (4.69)
Not Available	2 (1.56)
Pathological, N, *n* (%)	
N0	53 (41.41)
N1	18 (14.06)
N2	44 (34.38)
NX	11 (8.59)
Not Available	2 (1.56)
Pathological, M, *n* (%)	
M0	43 (33.59)
MX	13 (10.16)
Not Available	72 (56.25)

Data are expressed as either frequency with percentages or means ± standard deviations; T: tumor; N: node; M: metastasis.

**Table 2:** The areas under the receiver operating characteristic curve and diagnostic values of five differentially expressed miRNAs.

miRNAs	AUC	95% CI	Cutoff	Sensitivity (%)	Specificity (%)	*p*-Value
miR-196a-1	0.9447	0.8991–0.9903	<2.319	84.62	92.97	<0.0001
miR-196b	0.9838	0.9661–1.001	<11.47	92.31	95.31	<0.0001
miR-450a-2	0.9303	0.8588–1.002	<1.284	84.62	93.75	<0.0001
miR-503	0.9549	0.9153–0.9946	<2.26	84.62	93.75	<0.0001
miR-877	0.9285	0.8791–0.9779	<0.8552	84.62	83.59	<0.0001

## Data Availability

Data will be made available on request.

## Supplementary Materials

The following supporting information can be downloaded at https://www.mdpi.com/article/10.3390/cimb46070402/s1: Table S1: Prediction of target genes of differentially expressed miR-196b; Table S2: GO analysis of target genes of miR-196b; Table S3: KEGG enrichment analysis of target genes of miR-196b. Figure S1: Heatmap for the identification of differentially expressed miRNAs. Figure S2: Receiver operating characteristic curve analysis of 13 differentially expressed miRNAs in oral squamous cell carcinoma of the tongue.
