# Supplementary figures and images for: The Potential MicroRNA Diagnostic Biomarkers in Oral Squamous Cell Carcinoma of the Tongue
Source: Curr Issues Mol Biol. 2024 Jul 1;46(7):6746–56. doi: 10.3390/cimb46070402 (PMC11276561; doi:10.3390/cimb46070402)

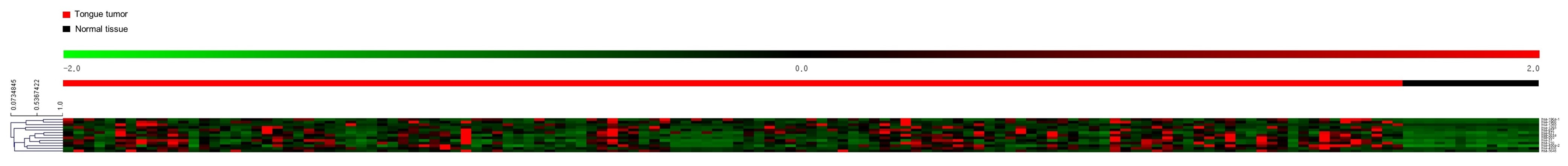

Supplement: Supplementary file 1 [file cimb-46-00402-s001.zip › Supplementary figure_S1.tif]

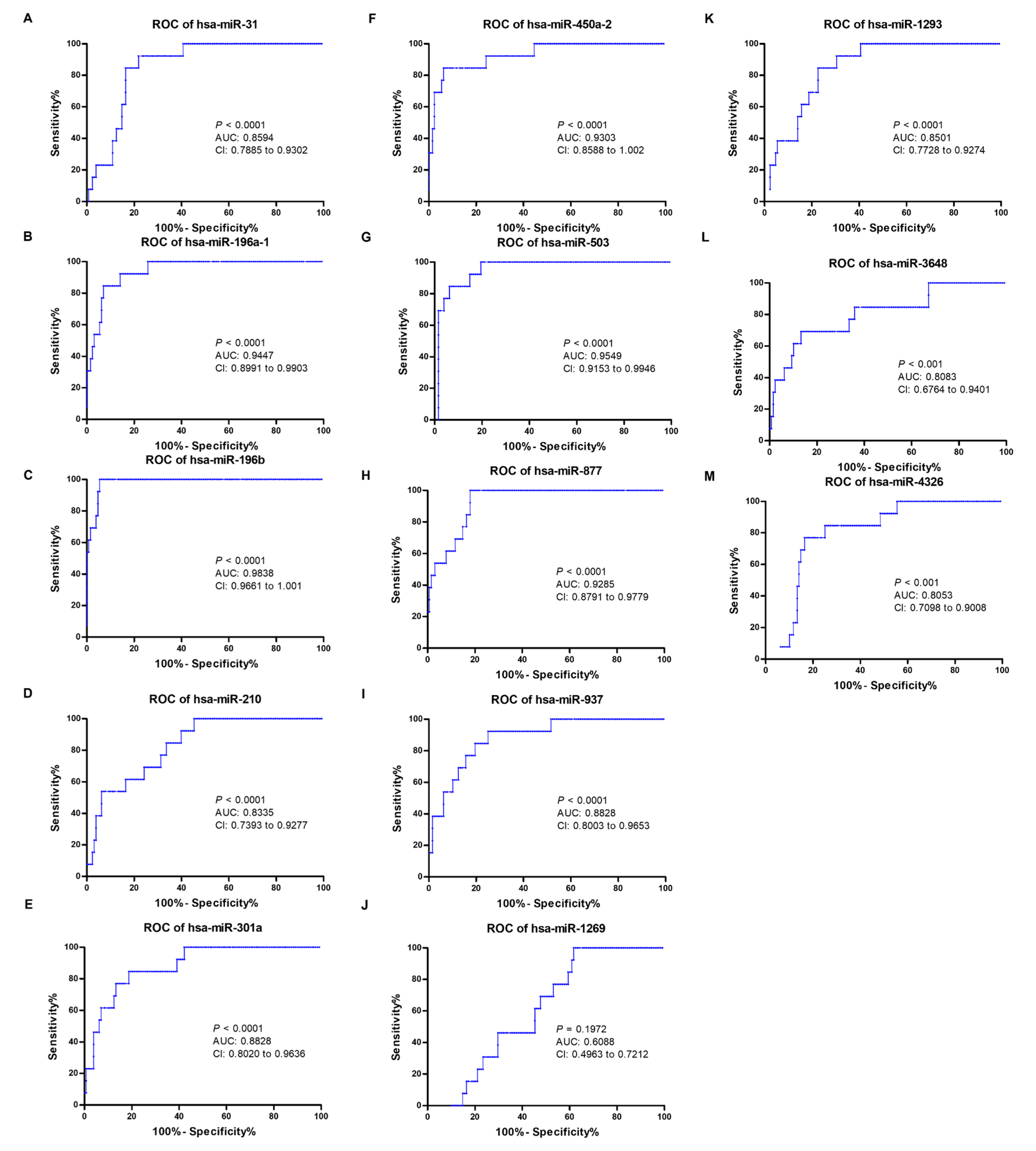

Supplement: Supplementary file 1 [file cimb-46-00402-s001.zip › Supplementary figure_S2.tif]
